# Current Cigarette Smoking Among Adults — United States, 2005–2012

**Published:** 2014-01-17

**Authors:** Israel T. Agaku, Brian A. King, Shanta R. Dube

**Affiliations:** 1 EIS officer, CDC; 2 Office on Smoking and Health, National Center for Chronic Disease Prevention and Health Promotion, CDC

Despite significant declines during the past 30 years, cigarette smoking among adults in the United States remains widespread, and year-to-year decreases in prevalence have been observed only intermittently in recent years (1,2). To assess progress made toward the *Healthy People 2020* objective of reducing the proportion of U.S. adults who smoke cigarettes to ≤12% (objective TU-1.1),* this report provides the most recent national estimates of smoking prevalence among adults aged ≥18 years, based on data from the 2012 National Health Interview Survey (NHIS). The findings indicate that the proportion of U.S. adults who smoke cigarettes fell to 18.1% in 2012. Moreover, during 2005–2012, the percentage of ever smokers who quit increased significantly, from 50.7% to 55.0%, and the proportion of daily smokers who smoked ≥30 cigarettes per day (CPD) declined significantly, from 12.6% to 7.0%. Proven population-level interventions, including tobacco price increases, high-impact antitobacco mass media campaigns, comprehensive smoke-free laws, and barrier-free access to help quitting, are critical to decreasing cigarette smoking and reducing the health and economic burden of tobacco-related diseases in the United States (3).

NHIS is an annual, nationally representative, in-person survey of the noninstitutionalized U.S. civilian population. Questions about cigarette smoking are directed to one randomly selected adult from each surveyed family. In 2012, a total of 34,525 adults aged ≥18 years were selected and participated, yielding a 61.2% response rate. Current smokers were respondents who reported smoking ≥100 cigarettes during their lifetime and, at the time of interview, reported smoking every day or some days. Former smokers were respondents who reported smoking ≥100 cigarettes during their lifetime but currently did not smoke. The mean number of CPD was calculated among daily current smokers. A quit attempt was defined as a report by a current smoker that they stopped smoking for >1 day during the preceding year because they were trying to quit smoking, or a report by a former smoker that they quit smoking during the preceding year.† Quit ratios were defined as the ratio of former smokers to ever smokers.

Data were adjusted for nonresponse and weighted to provide nationally representative estimates. Current smoking was assessed overall and by sex, age, race/ethnicity, education, poverty status,§ U.S. Census region,¶ and disability/limitation status.** Differences between groups were assessed using the chi-squared statistic and 95% confidence intervals. Quit ratios were calculated overall and by age group. Logistic regression was used to analyze overall trends in prevalence, CPD, and quit ratios during 2005–2012, controlling for sex, age, and race/ethnicity. The Wald test was used to determine statistical significance of trends from 2005 to 2012 (p<0.05).

In 2012, an estimated 18.1% (42.1 million) of U.S. adults were current cigarette smokers. Of these, 78.4% (33.0 million) smoked every day, and 21.6% (9.1 million) smoked some days. Overall smoking prevalence declined from 20.9% in 2005 to 18.1% in 2012 (p<0.05 for trend) (Table). In 2012, prevalence was significantly higher among males (20.5%) than females (15.8%) and among persons aged 18–24 years (17.3%), 25–44 years (21.6%), and 45–64 years (19.5%) than among those aged ≥65 years (8.9%). By race/ethnicity, prevalence was highest among respondents reporting multiple races (26.1%) and lowest among Asians (10.7%). By education, prevalence was highest among persons with a graduate education development certificate (41.9%) and lowest among those with a graduate (5.9%) or undergraduate (9.1%) degree. Prevalence was significantly higher among persons living below the poverty level (27.9%) than those living at or above this level (17.0%). By U.S. Census region, prevalence was significantly higher in the South (19.7%) and Midwest (20.6%) than the West (14.2%) and Northeast (16.5%). Respondents who reported having a disability/limitation with activities of daily living (disability/limitation) had a significantly higher prevalence (22.7%) than those with no disability/limitation (16.5%).

Among daily smokers, declines in mean CPD occurred from 16.7 in 2005 to 14.6 in 2012 (p<0.05 for trend). During 2005–2012, increases occurred in the proportion of daily smokers who smoked 1–9 CPD (16.4% to 20.8%) and 10–19 CPD (36.0% to 41.2%), whereas declines occurred in those smoking 20–29 CPD (34.9% to 31.0%) and ≥30 CPD (12.6% to 7.0%) (Figure 1) (p<0.05 for trend).

Among current smokers and former smokers who quit during the preceding year, 52.9% had made a quit attempt for >1 day. The overall quit ratio (i.e., the ratio of former to ever smokers) increased from 50.7% in 2005 to 55.0% in 2012 (Figure 2) (p<0.05). Quit ratios were lowest among adults aged 18–24 years and highest among those aged ≥65 years in each survey year. During 2005–2012, the largest increase in quit ratios (22.7% to 26.5% [p<0.05]) and decline in smoking prevalence (24.4% to 17.3% [p<0.05]) occurred among those aged 18–24 years.

## Editorial Note

During 2005–2012, cigarette smoking prevalence declined among U.S. adults, and the quit ratio (i.e., the percentage of ever smokers who had quit) increased. During the same period, the proportion of daily smokers who smoked ≥30 CPD also declined. Adults aged 18–24 years had the greatest decrease in cigarette smoking prevalence; however, this decline might be attributable, in part, to the use of other tobacco products, such as flavored little cigars, which are especially popular with this age group (4).

The decline in overall smoking prevalence from 20.9% in 2005 to 18.1% in 2012 is encouraging and likely reflects the success of tobacco control efforts across the country. However, given the slowing decline in adult smoking in recent years, continued implementation of evidence-based interventions outlined in the World Health Organization MPOWER package is critical.†† These include increasing the price of tobacco products, implementing and enforcing comprehensive smoke-free laws, warning about the dangers of tobacco use with antismoking media campaigns, and increasing access to help quitting. Such population-based interventions have been shown to reduce population smoking prevalence (3).

What is already known about this topic?

Approximately one in five U.S. adults smoke cigarettes, and certain population groups have a higher prevalence of smoking. Despite significant declines during the past 30 years, cigarette smoking among adults in the United States remains widespread, and year-to-year decreases in prevalence have been observed only intermittently in recent years.

What is added by this report?

Overall smoking prevalence declined significantly during 2005–2012 (from 20.9% to 18.1% [p<0.05]). In addition, among daily smokers, the average number of cigarettes smoked per day declined from 16.7 in 2005 to 14.6 in 2012 (p<0.05 for trend). During the same period, the largest increase in the percentage of ever smokers who quit (from 22.7% to 26.5% [p<0.05]) and the largest declines in smoking prevalence (from 24.4% to 17.3% [p<0.05]) were observed among persons aged 18–24 years.

What are the implications for public health practice?

Effective public health interventions that can continue progress toward meeting the *Healthy People 2020* target to reduce U.S. adult cigarette smoking to ≤12% include a combination of tobacco price increases, high-impact antitobacco mass media campaigns, comprehensive smoke-free laws, and barrier-free access to help quitting.

In recent years, major advances were made in tobacco control. These include the 2009 Family Smoking Prevention and Tobacco Control Act, which granted the Food and Drug Administration the authority to regulate the manufacture, distribution, and marketing of tobacco products.§§ Additionally, the 2009 Children’s Health Insurance Program Reauthorization Act¶¶ raised the federal tax rate for cigarettes from $0.39 to $1.01 per pack, and the 2010 Patient Protection and Affordable Care Act*** provided expanded coverage for evidence-based smoking-cessation treatments for many persons in the United States. Finally, in 2012, CDC debuted Tips from Former Smokers (TIPS),††† the first federally funded, nationwide, paid-media tobacco education campaign in the United States. During the campaign, calls to the quitline portal 1-800-QUIT-NOW increased 132%, and the number of unique visitors to a smoking cessation website (http://www.smokefree.gov) increased 428% (5). Additionally, an estimated 1.6 million quit attempts were attributable to the campaign (6).

Disparities in smoking prevalence described in this report are consistent with previous studies (2). Variations across racial/ethnic groups might be attributable, in part, to targeted tobacco product marketing or differences in the social acceptability of smoking, whereas disparities by education might be related to differences in understanding of the health hazards of smoking and increased vulnerability to tobacco marketing. Differences by disability/limitation status might be attributable, in part, to smoking-attributable disability in smokers and increased stress associated with disabilities (7). The high smoking prevalence observed among some population groups underscores the need for enhanced implementation and reach of proven strategies to prevent and reduce tobacco use among these groups.

The findings in this report are subject to at least six limitations. First, smoking status was self-reported and not validated by biochemical testing. However, self-reported smoking status correlates highly with serum cotinine levels (8). Second, small sample sizes for certain population groups resulted in less precise estimates. Third, data could not be disaggregated for specific racial/ethnic subgroups; although smoking prevalence was lowest among Hispanics and non-Hispanic Asians, variability in smoking prevalence exists among Hispanic and Asian subpopulations (9). Fourth, because NHIS does not include institutionalized populations and persons in the military, results might not be generalizable to these groups. Fifth, the NHIS response rate of 61.2% might have resulted in nonresponse bias, even after adjustment for nonresponse. Finally, these estimates might differ from those derived from other surveillance systems. For example, the National Survey on Drug Use and Health consistently yields higher current smoking estimates than NHIS (10). These differences can be explained, in part, by the varying survey methodologies, the types of surveys administered, and the definitions of current smoking that are used. However, trends in prevalence are comparable across surveys.

Sustained, comprehensive state tobacco control programs funded at CDC-recommended levels accelerate progress toward reducing the health burden and economic impact of tobacco-related diseases in the United States (3). However, during 2013, despite combined revenue of $25.7 billion from settlement payments and tobacco taxes for all states, only $459.5 million (1.8%) was spent on state comprehensive tobacco control programs, representing only 12.4% of the CDC-recommended level of funding for all states combined; moreover, only two states (Alaska and North Dakota) currently fund tobacco control programs at CDC-recommended levels.§§§ Implementation of comprehensive tobacco control policies and programs can result in a substantial reduction in tobacco-related morbidity and mortality and billions of dollars in savings from averted medical costs (3).

## Figures and Tables

**FIGURE 1:**
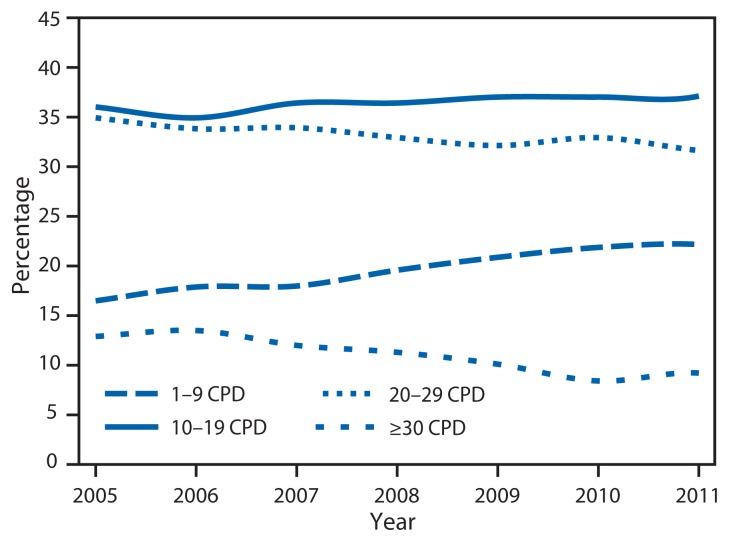
Percentage of daily smokers* aged ≥18 years, by number of cigarettes smoked per day (CPD) — National Health Interview Survey, United States, 2005–2012 * Persons who reported smoking ≥100 cigarettes during their lifetime and who, at the time of the survey, reported smoking cigarettes every day.

**FIGURE 2:**
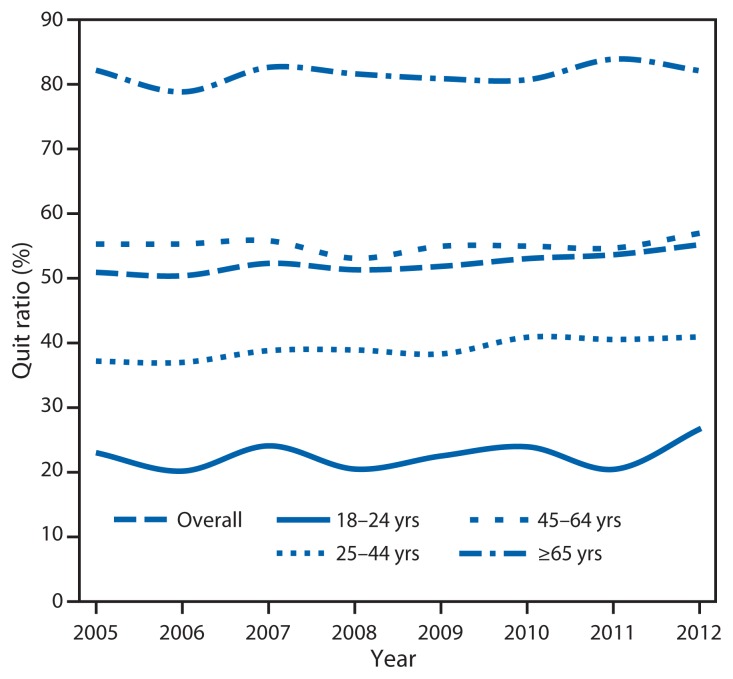
Quit ratios* among ever smokers^†^ aged ≥18 years, overall and by age group — National Health Interview Survey, United States, 2005–2012 * Defined as the ratio of former smokers to ever smokers for each survey year. ^†^ Persons who reported smoking ≥100 cigarettes during their lifetime.

**TABLE:** Percentage of persons aged ≥18 years who were current cigarette smokers,* by selected characteristics — National Health Interview Survey, United States, 2005 and 2012

Characteristic	Men				Women				Total			
		
	2005 (n = 13,762)		2012 (n = 15,273)		2005 (n = 17,666)		2012 (n = 19,252)		2005 (N = 31,428)		2012 (N = 34,525)	
					
	%	(95% CI)	%	(95% CI)	%	(95% CI)	%	(95% CI)	%	(95% CI)	%	(95% CI)
**Overall**	**23.9**	**(22.9–24.9)**	**20.5**	**(19.6–21.4)**	**18.1**	**(17.4–18.8)**	**15.8**	**(15.1–16.5)**	**20.9**	**(20.3–21.5)**	**18.1**	**(17.5–18.7)**
**Age group (yrs)**												
18–24	28.0	(25.0–31.0)	20.1	(17.1–23.1)	20.7	(18.3–23.1)	14.5	(12.3–16.7)	24.4	(22.4–26.4)	17.3	(15.5–19.2)
25–44	26.8	(25.4–28.3)	25.4	(23.8–27.1)	21.4	(20.2–22.6)	17.8	(16.6–19.0)	24.1	(23.1–25.1)	21.6	(20.5–22.7)
45–64	25.2	(23.7–26.7)	20.2	(18.8–21.6)	18.8	(17.7–19.9)	18.9	(17.6–20.2)	21.9	(21.0–22.8)	19.5	(18.6–20.5)
≥65	8.9	(7.6–10.2)	10.6	(9.3–12.0)	8.3	(7.3–9.3)	7.5	(6.6–8.5)	8.6	(7.8–9.4)	8.9	(8.1–9.7)
**Race/Ethnicity** †												
White	24.0	(22.8–25.2)	21.1	(19.9–22.2)	20.0	(19.1–20.9)	18.4	(17.4–19.3)	21.9	(21.1–22.7)	19.7	(18.9–20.4)
Black	26.7	(23.9–29.5)	22.1	(19.9–24.4)	17.3	(15.6–19.0)	14.8	(13.2–16.3)	21.5	(19.9–23.1)	18.1	(16.7–19.4)
Hispanic	21.1	(19.2–23.0)	17.2	(15.2–19.2)	11.1	(9.8–12.4)	7.8	(6.6–8.9)	16.2	(15.0–17.4)	12.5	(11.3–13.7)
American Indian/Alaska Native	37.5	(20.7–54.3)	25.5	(15.5–35.6)	26.8	(15.5–38.1)	18.7	(9.3–28.0)	32.0	(22.3–41.7)	21.8	(15.0–28.6)
Asian§	20.6	(15.7–25.5)	16.7	(13.7–19.8)	6.1	(3.7–8.5)	5.5	(4.0–7.0)	13.3	(10.4–16.2)	10.7	(9.1–12.3)
Multiple race	26.1	(16.3–35.9)	28.6	(21.0–36.3)	23.5	(14.8–32.2)	23.9	(17.6–30.2)	24.8	(17.7–31.9)	26.1	(21.3–31.0)
**Education** ¶												
0–12 years (no diploma)	29.5	(27.2–31.8)	29.5	(26.9–32.0)	21.9	(20.1–23.7)	20.2	(18.0–22.3)	25.5	(24.0–27.0)	24.7	(23.0–26.4)
8th grade or less	21.0	(17.7–24.3)	20.2	(16.9–23.4)	13.4	(11.1–15.7)	10.6	(8.2–13.1)	17.1	(15.1–19.1)	15.2	(13.2–17.3)
9–11th grade	36.8	(33.3–40.3)	38.5	(34.2–42.8)	29.0	(26.1–31.9)	26.4	(23.1–29.8)	32.6	(30.3–34.9)	32.1	(29.4–34.9)
12th grade, no diploma	30.2	(23.5–36.9)	25.5	(20.0–31.1)	22.2	(16.9–27.5)	23.7	(17.9–29.6)	26.0	(21.8–30.2)	24.7	(20.6–28.7)
GED	47.5	(41.4–53.6)	45.8	(39.6–51.9)	38.8	(33.6–44.0)	37.5	(31.6–43.3)	43.2	(39.0–47.4)	41.9	(37.5–46.4)
High school graduate	28.8	(27.0–30.6)	27.0	(25.0–29.0)	20.7	(19.3–22.1)	19.5	(17.8–21.2)	24.6	(23.5–25.7)	23.1	(21.8–24.5)
Some college, no diploma	26.2	(24.4–28.0)	22.6	(20.4–24.8)	19.5	(18.0–21.0)	19.4	(17.6–21.2)	22.5	(21.4–23.6)	20.9	(19.4–22.4)
Associate degree	26.1	(23.3–28.9)	18.7	(16.2–21.3)	17.1	(15.0–19.2)	17.2	(15.2–19.3)	20.9	(19.2–22.6)	17.9	(16.2–19.6)
Undergraduate degree	11.9	(10.5–13.3)	10.0	(8.5–11.4)	9.6	(8.3–10.9)	8.3	(7.1–9.6)	10.7	(9.8–11.6)	9.1	(8.1–10.1)
Graduate degree	6.9	(5.3–8.5)	6.3	(4.7–7.9)	7.4	(6.0–8.8)	5.5	(4.4–6.6)	7.1	(6.0–8.2)	5.9	(5.0–6.9)
**Poverty status** **												
At or above poverty level	23.7	(22.6–24.8)	19.1	(18.1–20.1)	17.6	(16.7–18.5)	15.0	(14.1–15.9)	20.6	(19.9–21.3)	17.0	(16.4–17.7)
Below poverty level	34.3	(31.1–37.5)	33.9	(31.0–36.9)	26.9	(24.5–29.3)	23.5	(21.5–25.5)	29.9	(27.9–31.9)	27.9	(26.2–29.6)
Unspecified	21.2	(19.2–23.2)	15.9	(13.5–18.2)	16.1	(14.8–17.4)	11.8	(10.1–13.4)	18.4	(17.2–19.6)	13.6	(12.2–15.0)
**U.S. Census region** ††												
Northeast	20.7	(18.6–22.8)	17.3	(15.3–19.4)	17.9	(16.3–19.5)	15.7	(13.9–17.6)	19.2	(17.8–20.6)	16.5	(15.1–17.9)
Midwest	27.3	(25.3–29.3)	22.7	(20.8–24.5)	21.3	(19.8–22.8)	18.6	(17.1–20.1)	24.2	(23.0–25.4)	20.6	(19.4–21.8)
South	25.3	(23.6–27.0)	22.4	(20.8–24.0)	18.5	(17.3–19.7)	17.2	(15.9–18.4)	21.8	(20.6–23.0)	19.7	(18.6–20.7)
West	20.1	(18.3–21.9)	17.7	(16.1–19.4)	13.9	(12.6–15.2)	10.8	(9.5–12.1)	17.0	(16.0–18.0)	14.2	(13.1–15.3)
**Disability/Limitation** §§												
Any disability/limitation	—¶¶	—¶¶	25.5	(22.7–28.4)	—¶¶	—¶¶	20.3	(17.9–22.8)	—¶¶	—¶¶	22.7	(20.9–24.4)
No disability/limitation	—¶¶	—¶¶	18.6	(17.4–19.9)	—¶¶	—¶¶	14.5	(13.5–15.5)	—¶¶	—¶¶	16.5	(15.7–17.3)

**Abbreviations:** CI = confidence interval; GED = General Education Development certificate.

* Persons who reported smoking at least 100 cigarettes during their lifetime and who, at the time of interview, reported smoking every day or some days. Excludes 296 (2005) and 269 (2012) respondents whose smoking status was unknown.

† Excludes 45 (2005) and 68 (2012) respondents of unknown race. Unless indicated otherwise, all racial/ethnic groups are non-Hispanic; Hispanics can be of any race.

§ Does not include Native Hawaiians or Other Pacific Islanders.

¶ Among persons aged ≥25 years. Excludes 339 (2005) and 112 (2012) persons whose educational level was unknown.

** Family income is reported by the family respondent who might or might not be the same as the sample adult respondent from whom smoking information is collected. 2005 estimates are based on reported family income and 2004 poverty thresholds published by the U.S. Census Bureau, and 2012 estimates are based on reported family income and 2011 poverty thresholds published by the U.S. Census Bureau.

†† *Northeast*: Connecticut, Maine, Massachusetts, New Hampshire, New Jersey, New York, Pennsylvania, Rhode Island, and Vermont. *Midwest*: Illinois, Indiana, Iowa, Kansas, Michigan, Minnesota, Missouri, Nebraska, North Dakota, Ohio, South Dakota, and Wisconsin. *South*: Alabama, Arkansas, Delaware, District of Columbia, Florida, Georgia, Kentucky, Louisiana, Maryland, Mississippi, North Carolina, Oklahoma, South Carolina, Tennessee, Texas, Virginia, and West Virginia. *West*: Alaska, Arizona, California, Colorado, Hawaii, Idaho, Montana, Nevada, New Mexico, Oregon, Utah, Washington, and Wyoming.

§§ Disability defined based on self-reported presence of selected impairments, including vision, hearing, cognition, and movement. Limitations in performing activities of daily living defined based on response to the question, “Because of a physical, mental, or emotional problem, does [person] need the help of other persons with personal care needs, such as eating, bathing, dressing, or getting around inside this home?” Limitations in performing instrumental activities of daily living defined based on response to the question, “Because of a physical, mental, or emotional problem, does [person] need the help of other persons in handling routine needs, such as everyday household chores, doing necessary business, shopping, or getting around for other purposes?” Any disability/limitation defined as a “yes” response pertaining to at least one of the disabilities/limitations listed (i.e., vision, hearing, cognition, movement, activities of daily living, or instrumental activities of daily living).

¶¶ Questions pertaining to disabilities/limitations were not included in the 2005 National Health Interview Survey.

## Footnotes

* Additional information available at http://healthypeople.gov/2020/topicsobjectives2020.

† Additional information available at http://www.healthindicators.gov/indicators/smoking-cessation-attempts-by-adult-smokers-percent_1513/profile.

§ Based on reported family income and 2011 poverty thresholds published by the U.S. Census Bureau.

¶ *Northeast:* Connecticut, Maine, Massachusetts, New Hampshire, New Jersey, New York, Pennsylvania, Rhode Island, and Vermont. *Midwest:* Illinois, Indiana, Iowa, Kansas, Michigan, Minnesota, Missouri, Nebraska, North Dakota, Ohio, South Dakota, and Wisconsin. *South:* Alabama, Arkansas, Delaware, District of Columbia, Florida, Georgia, Kentucky, Louisiana, Maryland, Mississippi, North Carolina, Oklahoma, South Carolina, Tennessee, Texas, Virginia, and West Virginia. *West:* Alaska, Arizona, California, Colorado, Hawaii, Idaho, Montana, Nevada, New Mexico, Oregon, Utah, Washington, and Wyoming.

** Disability defined based on self-reported presence of selected impairments, including vision, hearing, cognition, and movement. Limitations in performing activities of daily living defined based on response to the question, “Because of a physical, mental, or emotional problem, does [person] need the help of other persons with personal care needs, such as eating, bathing, dressing, or getting around inside this home?” Limitations in performing instrumental activities of daily living defined based on response to the question, “Because of a physical, mental, or emotional problem, does [person] need the help of other persons in handling routine needs, such as everyday household chores, doing necessary business, shopping, or getting around for other purposes?” Any disability/limitation defined as a “yes” response pertaining to at least one of the disabilities/limitations listed (i.e., vision, hearing, cognition, movement, activities of daily living, or instrumental activities of daily living).

†† Additional information available at http://www.who.int/tobacco/mpower/mpower_report_full_2008.pdf.

§§ Additional information available at http://www.fda.gov/tobaccoproducts.

¶¶ Additional information available at http://www.cms.gov/chipra.

*** Additional information available at http://www.dol.gov/ebsa/healthreform.

††† Additional information available at http://www.cdc.gov/tobacco/campaign/tips.

§§§ Robert Wood Johnson Foundation. Broken promises to our children: the 1998 state tobacco settlement fourteen years later. A report on the states’ allocation of the tobacco settlement dollars. Princeton, NJ: Robert Wood Johnson Foundation; 2012. Available at http://www.tobaccofreekids.org/content/what_we_do/state_local_issues/settlement/FY2013/1.%202012%20State%20Report%20-%20Full.pdf.
